# Surveillance Immunity: An Emerging Paradigm of Innate Defense Activation in *Caenorhabditis elegans*

**DOI:** 10.1371/journal.ppat.1005795

**Published:** 2016-09-15

**Authors:** Read Pukkila-Worley

**Affiliations:** Program in Innate Immunity, Division of Infectious Diseases and Immunology, University of Massachusetts Medical School, Worcester, Massachusetts, United States of America; The University of North Carolina at Chapel Hill, UNITED STATES

## Pathogen-Mediated Disruption of Host Physiology Leads to Immune Activation in *Caenorhabditis elegans*

**Fig 1:**
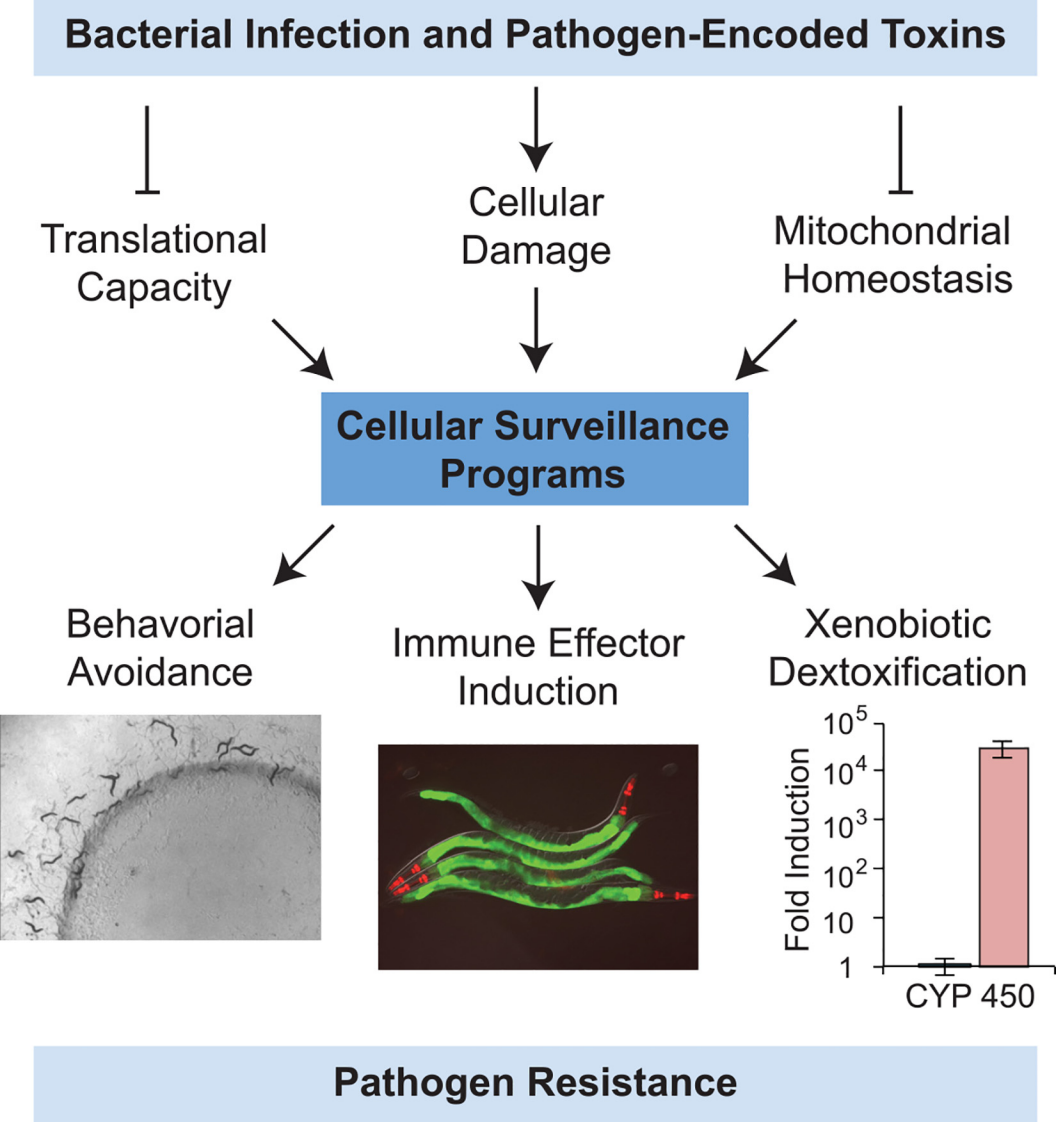
Immune surveillance in *C*. *elegans*. Cellular damage and disruptions in translational capacity or mitochondrial homeostasis that occur during microbial infection, or through the effects of pathogen-encoded toxins, are detected by surveillance programs to activate protective host responses in nematodes, examples of which are presented. The photograph on the left shows the behavioral avoidance phenotype of wild-type *C*. *elegans* to a xenobiotic toxin, which was placed in the lawn of nutritious and otherwise attractive bacteria. In the center, a transgenic *C*. *elegans* strain, which was engineered to express green fluorescent protein (GFP) as a visual readout of immune pathway activation, was photographed after exposure to an immunostimulatory anti-infective molecule. GFP stains the intestinal epithelial cells of these animals, the site where this immune pathway is strongly activated (the red color in the pharynx is a marker used to identify transgenic animals). On the right, quantitative real-time PCR data are presented to show the dramatic induction of a cytochrome P450 detoxification gene by a xenobiotic toxin. These images have been previously published [20,21] and are used here with permission.

The evolution of bacterivorous nematodes, such as *Caenorhabditis elegans*, has been shaped by interactions with environmental microbes, which for nematodes are both sources of food and agents of disease. As a result, *C*. *elegans* has evolved protective host responses coordinated through multiple pathways, which are required for host survival during microbial infection. Following exposure to a pathogen, putative immune effectors are transcriptionally up-regulated, which has led to an extensive search for the mechanisms underlying pathogen recognition in this simple metazoan host. Biological rationale for the existence of inducible immune defenses has come from the recognition that physiologic [1,2] or aberrant [3] activation of immune responses constitutes an important source of cellular stress for nematodes, arguing that these protective host responses must be tightly regulated to ensure host survival. However, despite much effort, the mechanisms underlying the activation and regulation of immune pathways in nematodes have, until recently, been elusive. In mammals, binding of conserved microbial molecules (so-called microbe-associated molecular patterns, or MAMPs) to cell surface pattern-recognition receptors (e.g., toll-like receptors) is a major method of pathogen detection. Such mechanisms may operate in nematodes [4–6], but a bona fide MAMP and its receptor have yet to be characterized in worms. Recently, a number of studies have supported the hypothesis that the nematode monitors for perturbations in host physiology that accompany infection with pathogenic microbes or the effects of their secreted toxins [7–9]. A related concept was originally pioneered in studies of plant immunity, where it is often called effector-triggered immunity. The major emerging theme here is that the mechanisms of surveillance immunity, as they are referred to in this review, are molded by the strategies employed by microbes to cause disease in the host (Fig 1).

## Inhibition of Host Translation Activates a Protective Immune Response in Nematodes

A multitude of bacteria produce toxins that interrupt host mRNA translation. In the case of *C*. *elegans*, this appears to have created selection pressure to evolve mechanisms that monitor overall translation capacity as a means to detect pathogen infection. Characterization of these mechanisms by three separate groups working in parallel provided the first demonstration of surveillance immunity in nematodes [7–9]. One such toxin that targets host translation is exotoxin A (ToxA), which is produced by the human bacterial pathogen *Pseudomonas aeruginosa* and cripples elongation of the growing peptide strand by ribosylating host elongation factor 2. Exposure of *C*. *elegans* to *P*. *aeruginosa* interrupts translation in intestinal epithelial cells [8]. This, in turn, causes an increase in the protein expression of a transcription factor called ZIP-2, which works together with the conserved regulator CEBP-2 to regulate immune responses in *C*. *elegans* [7,8,10]. The end result is the up-regulation of defense-associated genes via pathways that are required to survive the otherwise lethal effects of this toxin [7–9]. Importantly, nematodes respond to the inhibition of translation to induce this response rather than to the structure of the toxin itself or to the effects on its host target, EF2 [7]. Induction of immune defenses in nematodes also occurs if host translation is interrupted through mutation or RNA interference (RNAi)-mediated gene knockdown of genes required for protein synthesis, which the host may be interpreting as pathogen or toxin exposure [8,9]. Interestingly, reduction of translation (and interruption of other core cellular processes) induces a behavioral avoidance response in *C*. *elegans*, which is controlled by a neuroendocrine axis involving serotonergic and c-Jun N-terminal kinase (JNK) signaling [9]. Together, these studies indicate that the overall state of host translation is monitored as a means to engage protective host responses, which involve the elaboration of protective immune effectors and a behavioral avoidance response.

## Perturbation of Mitochondrial Homeostasis Leads to Immune Pathway Induction in *C*. *elegans*

Disruption of mitochondrial homeostasis often accompanies bacterial infection, which occurs at least in part through the direct effects of pathogen-encoded toxins that poison mitochondrial function [11]. Interestingly, 18% of 560 bacterial species isolated from natural habitats of *C*. *elegans* caused mitochondrial stress in the laboratory strain of *C*. *elegans*, which highlights the selection pressure faced by free-living nematodes [12]. Indeed, several studies have now established that the mechanisms, which function to maintain mitochondrial function under nonhomeostatic conditions, also engage innate immune defenses [8,12–14]. During mitochondrial stress, organelle function is maintained by nuclear-encoded molecular chaperones, whose transcription is regulated by a signaling pathway called the mitochondrial unfolded protein response (UPR^mt^) [14]. The transcription factor ATFS-1, a key regulator of the UPR^mt^, is normally taken efficiently into the mitochondria and degraded, but under conditions of mitochondrial stress, the uptake of ATFS-1 into mitochondria is compromised, freeing cytosolic ATFS-1 to traffic to the nucleus, where it induces mitochondrial stress-response proteins [14]. Interestingly, ATFS-1 also enters the nucleus during *P*. *aeruginosa* infection and causes the transcriptional induction of putative antibacterial immune effectors, which are required for *C*. *elegans* to resist infection by *P*. *aeruginosa* [13]. In addition, the lipid ceramide acts upstream of ATFS-1 in the coordination of protective host responses following disruption of mitochondrial function [12]. Likewise, genetic disruption of mitochondrial function, as with the inhibition of translation, induces a behavioral avoidance response, which is protective during microbial infection [9]. All together, these data nicely demonstrate that surveillance of mitochondrial function is another cue used by *C*. *elegans* to detect pathogen invasion.

## Other Examples and Extensions of the Surveillance Immunity Hypothesis

Intriguingly, several other examples of surveillance immunity have been described in *C*. *elegans*. Disruption of the ubiquitin proteasome system, which targets proteins for degradation, leads to immune effector activation [9,15]. In addition, DNA damage in the gonad confers resistance to subsequent bacterial infection, perhaps via a mechanism that involves monitoring the integrity of the genome as a means to mount protective immune responses [16]. Likewise, disruption of histone-related processes also leads to immune effector activation [8,9].

Another key insight into the mechanisms of pathogen detection in *C*. *elegans* has come from the recognition that host-derived signals of cellular damage are potent activators of immune responses. In mammals, these immune response elicitors are called damage-associated molecular patterns (DAMPs). A tyrosine derivative, hydroxyphenyllactic acid (HPLA), accumulates in *C*. *elegans* following infection with *Drechmeria coniospora*, a fungal pathogen that first attacks the extracellular cuticle of nematodes and evokes a potent antifungal immune response in the epidermis. HPLA is recognized by the epidermal G protein-coupled receptor DCAR-1 to activate immune defenses, which is required to resist killing by *D*. *coniospora* [17]. The induction of immune effector expression by DCAR-1 can also be elicited by physical wounding of the epidermis, arguing that this receptor controls a DAMP-mediated response in nematodes. In a separate study, physical injury was also found to elicit antifungal immune responses in the *C*. *elegans* epidermis [18]. Disruption of structures called hemidesmosomes, which anchor epidermal cells to the extracellular cuticle, trigger expression of antifungal immune effectors by liberating a transcription factor, STA-1, which is normally associated with these proteins [18]. Thus, in addition to monitoring cellular homeostasis as a means to detect pathogen invasion, nematodes also survey for infection-induced cellular damage. Indeed, future studies may find that secondary signals elaborated following pathogen-mediated disruption of core physiological processes are detected to activate immune defenses, thereby connecting the DAMP and surveillance immunity hypotheses.

## Integration of Protective Host Responses following Perturbations in Host Physiology

An extension of the immune surveillance hypothesis involves the host response to chemical toxins, which often poison the same cellular processes as pathogen-encoded effectors. As in mammals, *C*. *elegans* possess a suite of inducible genes, including cytochrome P450s and glutathione-s-transferases, that metabolize toxins [19]. Interestingly, disruption of core physiological processes by RNAi or through genetic mutation causes the induction of these small molecule detoxification enzymes, as well as genes involved in the defense response to pathogens [7–9]. In addition, a conserved subunit of the Mediator transcriptional regulatory complex, MDT-15, links the induction of innate immune defenses and the up-regulation of xenobiotic detoxification genes, perhaps as a means to counter both pathogen infection and the effects of microbial toxins [20,21]. Moreover, interruption of host translation in the germline leads to the induction of small molecule detoxification genes, in addition to innate immune effectors [22]. The up-regulation of detoxification responses in this context requires the action of lipid biosynthesis enzymes, which presumably synthesize a soluble signal that is sensed in the soma to coordinate this protective host response in a cell nonautonomous manner. Together, these data indicate that core cellular processes are monitored as a means to mount protective host responses towards both biotic and abiotic intoxication.

## References

[1] Richardson CE, Kooistra T, Kim DH. An essential role for XBP-1 in host protection against immune activation in *C*. *elegans* . Nature. 2010;463: 1092–1095. 10.1038/nature08762 20182512 PMC2834299

[2] Richardson CE, Kinkel S, Kim DH. Physiological IRE-1-XBP-1 and PEK-1 signaling in *Caenorhabditis elegans* larval development and immunity. PLoS Genet. 2011;7: e1002391 10.1371/journal.pgen.1002391 22125500 PMC3219621

[3] Cheesman HK, Feinbaum RL, Thekkiniath J, Dowen RH, Conery AL, Pukkila-Worley R. Aberrant activation of p38 MAP kinase-dependent innate immune responses is toxic to *Caenorhabditis elegans* . G3 (Bethesda). 2016;6: 541–549. 26818074 10.1534/g3.115.025650 PMC4777117

[4] Pukkila-Worley R, Ausubel FM, Mylonakis E. *Candida albicans* infection of *Caenorhabditis elegans* induces antifungal immune defenses. PLoS Pathog. 2011;7: e1002074 10.1371/journal.ppat.1002074 21731485 PMC3121877

[5] Irazoqui JE, Troemel ER, Feinbaum RL, Luhachack LG, Cezairliyan BO, Ausubel FM. Distinct pathogenesis and host responses during infection of *C*. *elegans* by *P*. *aeruginosa* and *S*. *aureus* . PLoS Pathog. 2010;6: e1000982 10.1371/journal.ppat.1000982 20617181 PMC2895663

[6] Twumasi-Boateng K, Shapira M. Dissociation of immune responses from pathogen colonization supports pattern recognition in *C*. *elegans* . PLoS ONE. 2012;7: e35400 10.1371/journal.pone.0035400 22514739 PMC3325959

[7] McEwan DL, Kirienko NV, Ausubel FM. Host translational inhibition by *Pseudomonas aeruginosa* Exotoxin A triggers an immune response in *Caenorhabditis elegans* . Cell Host Microbe. 2012;11: 364–374. 10.1016/j.chom.2012.02.007 22520464 PMC3334877

[8] Dunbar TL, Yan Z, Balla KM, Smelkinson MG, Troemel ER. *C*. *elegans* detects pathogen-induced translational inhibition to activate immune signaling. Cell Host Microbe. 2012;11: 375–386. 10.1016/j.chom.2012.02.008 22520465 PMC3334869

[9] Melo JA, Ruvkun G. Inactivation of conserved *C*. *elegans* genes engages pathogen- and xenobiotic-associated defenses. Cell. 2012;149: 452–466. 10.1016/j.cell.2012.02.050 22500807 PMC3613046

[10] Reddy KC, Dunbar TL, Nargund AM, Haynes CM, Troemel ER. The *C*. *elegans* CCAAT-enhancer-binding protein gamma is required for surveillance immunity. Cell Rep. 2016;14: 1581–1589. 10.1016/j.celrep.2016.01.055 26876169 PMC4767654

[11] Kirienko NV, Ausubel FM, Ruvkun G. Mitophagy confers resistance to siderophore-mediated killing by *Pseudomonas aeruginosa* . Proc Natl Acad Sci USA. 2015;112: 1821–1826. 10.1073/pnas.1424954112 25624506 PMC4330731

[12] Liu Y, Samuel BS, Breen PC, Ruvkun G. *Caenorhabditis elegans* pathways that surveil and defend mitochondria. Nature. 2014;508: 406–410. 10.1038/nature13204 24695221 PMC4102179

[13] Pellegrino MW, Nargund AM, Kirienko NV, Gillis R, Fiorese CJ, Haynes CM. Mitochondrial UPR-regulated innate immunity provides resistance to pathogen infection. Nature. 2014;516: 414–417. 10.1038/nature13818 25274306 PMC4270954

[14] Nargund AM, Pellegrino MW, Fiorese CJ, Baker BM, Haynes CM. Mitochondrial import efficiency of ATFS-1 regulates mitochondrial UPR activation. Science. 2012;337: 587–590. 10.1126/science.1223560 22700657 PMC3518298

[15] Bakowski MA, Desjardins CA, Smelkinson MG, Dunbar TA, Lopez-Moyado IF, Rifkin SA, et al Ubiquitin-mediated response to microsporidia and virus infection in *C*. *elegans* . PLoS Pathog. 2014;10: e1004200 10.1371/journal.ppat.1004200 24945527 PMC4063957

[16] Ermolaeva MA, Segref A, Dakhovnik A, Ou H-L, Schneider JI, Utermöhlen O, et al DNA damage in germ cells induces an innate immune response that triggers systemic stress resistance. Nature. 2013;501: 416–420. 10.1038/nature12452 23975097 PMC4120807

[17] Zugasti O, Bose N, Squiban B, Belougne J, Kurz CL, Schroeder FC, et al Activation of a G protein-coupled receptor by its endogenous ligand triggers the innate immune response of *Caenorhabditis elegans* . Nat Immunol. 2014;15: 833–838. 10.1038/ni.2957 25086774 PMC4139443

[18] Zhang Y, Li W, Li L, Li Y, Fu R, Zhu Y, et al Structural damage in the *C*. *elegans* epidermis causes release of STA-2 and induction of an innate immune response. Immunity. 2015;42: 309–320. 10.1016/j.immuni.2015.01.014 25692704

[19] McElwee JJ, Schuster E, Blanc E, Thomas JH, Gems D. Shared transcriptional signature in *Caenorhabditis elegans* dauer larvae and long-lived *daf-2* mutants implicates detoxification system in longevity assurance. J Biol Chem. 2004;279: 44533–44543. 15308663 10.1074/jbc.M406207200

[20] Pukkila-Worley R, Feinbaum RL, McEwan DL, Conery AL, Ausubel FM. The evolutionarily conserved mediator subunit MDT-15/MED15 links protective innate immune responses and xenobiotic detoxification. PLoS Pathog. 2014;10: e1004143 10.1371/journal.ppat.1004143 24875643 PMC4038581

[21] Pukkila-Worley R, Feinbaum R, Kirienko NV, Larkins-Ford J, Conery AL, Ausubel FM. Stimulation of host immune defenses by a small molecule protects *C*. *elegans* from bacterial infection. PLoS Genet. 2012;8: e1002733 10.1371/journal.pgen.1002733 22719261 PMC3375230

[22] Govindan JA, Jayamani E, Zhang X, Breen P, Larkins-Ford J, Mylonakis E, et al Lipid signalling couples translational surveillance to systemic detoxification in *Caenorhabditis elegans* . Nat Cell Biol. 2015; 17:1294–1303. 10.1038/ncb3229 26322678 PMC4589496

